# Supplementary material for: SNP rs6564851 in the BCO1 Gene Is Associated with Varying Provitamin a Plasma Concentrations but Not with Retinol Concentrations among Adolescents from Rural Ghana
Source: Nutrients. 2020 Jun 16;12(6):1786. doi: 10.3390/nu12061786 (PMC7353293; doi:10.3390/nu12061786)
Supplement: Supplementary file 1 [file nutrients-12-01786-s001.pdf]

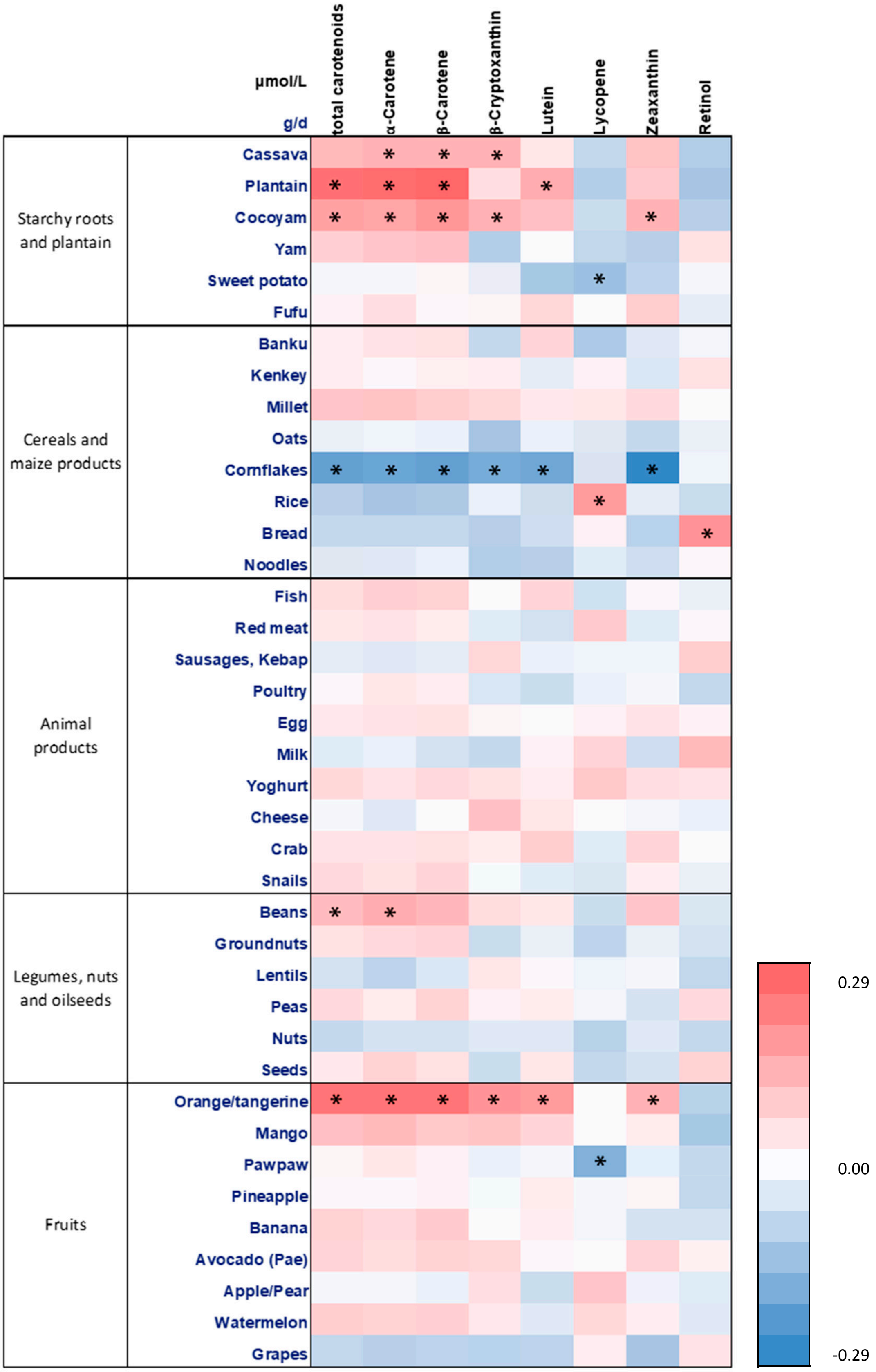

Figure S1. Spearman correlation of plasma carotenoid and retinol concentrations [µmol/L] with calculated intake [g/d] per food group; *$p<0.05$

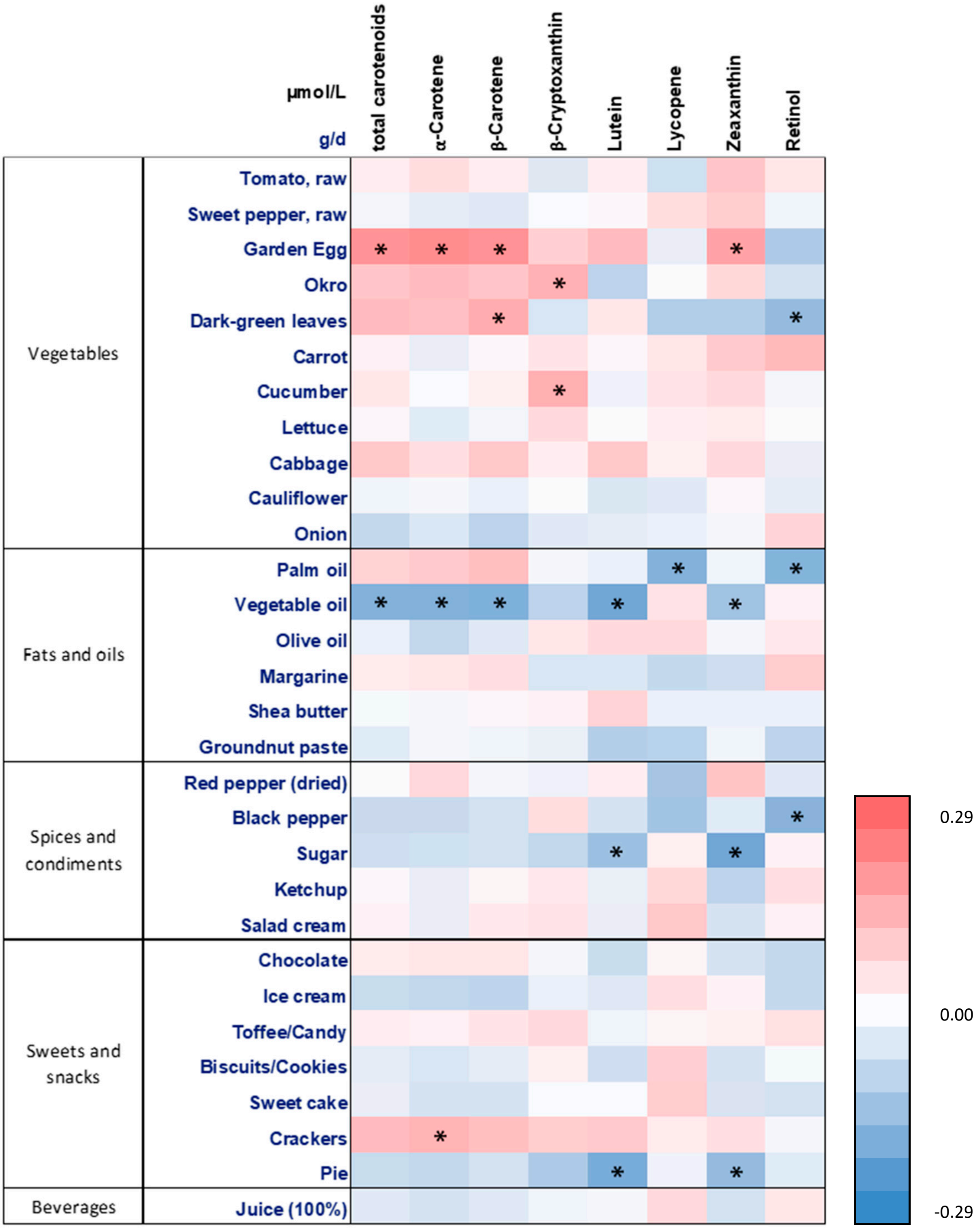

Figure S1 (continued). Spearman correlation of plasma carotenoid and retinol concentrations [µmol/L] with calculated intake [g/d] per food group; *p<0.05

Table S1. Concentrations of retinol, α-carotene, β-carotene, β-cryptoxanthin, lutein, lycopene and zeaxanthin analysed in Standard Reference Material® 968d provided by the National Institute of Standards and Technology (NIST).

	**NIST**	**Potsdam Lab**	
**Analyte**	**Concentration of the SRM 968d**	**Measured concentration** *(n = 5)*	**CV%**
**Retinol** (μmol/L)	1.09 ± 0.17	1.04 ± 0.04	3.88
**α-carotene** (μg/mL)	0.018 ± 0.004	0.008 ± 0.001	8.77
**β-carotene** (μg/mL)	0.145 ± 0.013	0.127 ± 0.007	5.27
**β-cryptoxanthin** (μg/mL)	0.050 ± 0.024	0.048 ± 0.002	3.63
**Lutein** (μg/mL)	0.085 ± 0.028	0.076 ± 0.007	9.50
**Lycopene** (μg/mL)	0.517 ± 0.063	0.440 ± 0.022	5.04
**Zeaxanthin** (μg/mL)	0.041 ± 0.009	0.032 ± 0.003	10.06

Data are presented as means ± standard deviations. CV, coefficient of variation.

nutrients

MDPI

Table S2. Concentrations of plasma total carotenoids, provitamin A and retinol, and vitamin A deficiency across genotypes of the variants in the BCO1 gene

	n	total carotenoids [µmol/L]	*p*-value	provitamin A [µmol/L]	*p*-value	retinol [µmol/L]	*p*-value	vitamin A deficient [%]	χ^2 *p*-value
**rs6564851**									
**TT**	89	2.59 (2.21-3.50)		2.03 (1.66-2.80)		0.77 (0.64-0.92)		37.1 (33)	
**GT**	82	3.22 (2.17-4.02)	0.111	2.69 (1.70-3.42)	0.110	0.77 (0.65-0.87)	0.748	35.4 (29)	0.753
**GG**	18	2.93 (2.16-3.80)		2.21 (1.68-3.27)		0.80 (0.62-0.96)		27.8 (5)	
**GT/GG**	100	3.07 (2.17-4.02)	0.042	2.48 (1.69-3.38)	0.042	0.77 (0.64-0.88)	0.719	34.0 (34)	0.659
**MAF**	0.31								
**rs7500996**									
**TT**	90	2.80 (2.10-3.78)		2.12 (1.66-3.27)		0.75 (0.62-0.90)		37.8 (34)	
**CT**	76	3.07 (2.24-3.93)	0.486	2.51 (1.68-3.27)	0.492	0.78 (0.67-0.91)	0.613	30.3 (23)	0.416
**CC**	23	2.62 (2.17-3.23)		2.02 (1.70-2.41)		0.79 (0.62-0.94)		43.5 (10)	
**CT/CC**	99	2.81 (2.23-3.83)	0.555	2.27 (1.70-3.23)	0.607	0.79 (0.65-0.92)	0.339	33.3 (33)	0.524
**MAF**	0.32								
**rs10048138**									
**GG**	71	3.00 (2.08-4.05)		2.24 (1.61-3.33)		0.76 (0.62-0.89)		35.2 (25)	
**AG**	92	2.77 (2.12-3.67)	0.708	2.11 (1.66-3.04)	0.631	0.78 (0.66-0.91)	0.822	32.6 (30)	0.443
**AA**	26	2.77 (2.31-3.98)		2.18 (1.88-3.45)		0.74 (0.64-0.94)		46.2 (12)	
**AG/AA**	118	2.77 (2.21-3.76)	0.744	2.14 (1.70-3.22)	0.828	0.77 (0.65-0.92)	0.533	36.6 (42)	0.958
**MAF**	0.38								

Table S2 (continued). Concentrations of plasma total carotenoids, provitamin A and retinol, and vitamin A deficiency across genotypes of the variants in the BCO1 gene

**rs6420424**									
**GG**	63	2.93 (2.21-3.78)		2.18 (1.66-3.22)		0.77 (0.64-0.94)		36.5 (23)	
**AG**	92	2.73 (2.09-3.78)	0.758	2.09 (1.63-3.24)	0.684	0.79 (0.67-0.92)	0.318	30.4 (28)	0.218
**AA**	34	2.87 (2.31-3.91)		2.33 (1.87-3.42)		0.72 (0.58-0.88)		47.1 (16)	
**AG/AA**	126	2.74 (2.16-3.80)	0.830	2.12 (1.66-3.26)	0.877	0.77 (0.64-0.91)	0.924	34.9 (44)	0.830
**MAF**	0.42								
**rs8044334**									
**TT**	35	2.75 (2.24-3.71)		2.27 (1.75-3.22)		0.74 (0.61-0.89)		32.9 (15)	
**GT**	91	2.81 (2.21-3.78)	0.957	2.13 (1.70-3.22)	0.984	0.78 (0.66-0.93)	0.392	31.9 (29)	0.502
**GG**	63	2.93 (2.05-4.05)		2.18 (1.45-3.50)		0.77 (0.64-0.90)		36.5 (23)	
**GT/GG**	154	2.83 (2.14-3.83)	0.897	2.14 (1.66-3.27)	0.918	0.78 (0.65-0.92)	0.190	33.8 (52)	0.310
**MAF**	0.43								

Continuous variables are presented as medians (interquartile ranges), categorical variables are shown as percentages (numbers)

Table S3. Plasma concentrations of provitamin A carotenoids across genotypes of the variants in the BCO1 gene

	n	*α*-carotene [μmol/L]	*p*-value	β-carotene [μmol/L]	*p*-value	β-cryptoxanthin [μmol/L]	*p*-value
**rs6564851**							
**TT**	89	0.56 (0.47-0.75)		1.40 (1.12-1.91)		0.09 (0.06-0.12)	
**GT**	82	0.69 (0.49-0.94)	0.085	1.88 (1.10-2.42)	0.086	0.10 (0.08-0.12)	0.127
**GG**	18	0.58 (0.46-0.81)		1.52 (1.19-2.29)		0.11 (0.08-0.14)	
**GT/GG**	100	0.66 (0.46-0.91)	0.053	1.73 (1.13-2.41)	0.029	0.10 (0.08-0.13)	0.075
**MAF**	0.31						
**rs7500996**							
**TT**	90	0.60 (0.46-0.87)		1.43 (1.12-2.28)		0.09 (0.06-0.12)	
**CT**	76	0.65 (0.46-0.90)	0.642	1.73 (1.11-2.22)	0.507	0.10 (0.07-0.13)	0.692
**CC**	23	0.55 (0.49-0.75)		1.37 (1.10-1.63)		0.09 (0.06-0.12)	
**CT/CC**	99	0.62 (0.47-0.86)	0.664	1.55 (1.10-2.14)	0.620	0.09 (0.07-0.13)	0.755
**MAF**	0.32						
**rs10048138**							
**GG**	71	0.60 (0.46-0.94)		1.55 (1.08-2.29)		0.10 (0.07-0.13)	
**AG**	92	0.61 (0.46-0.86)	0.795	1.46 (1.10-2.04)	0.577	0.09 (0.07-0.12)	0.567
**AA**	26	0.64 (0.50-0.83)		1.47 (1.27-2.40)		0.09 (0.06-0.12)	
**AG/AA**	118	0.61 (0.48-0.86)	0.943	1.46 (1.17-2.07)	0.747	0.09 (0.06-0.12)	0.310
**MAF**	0.38						

Table S3 (continued). Plasma concentrations of provitamin A carotenoids across genotypes of the variants in the BCO1 gene

**rs6420424**							
**GG**	63	0.62 (0.46-0.89)		1.50 (1.16-2.28)		0.09 (0.07-0.12)	0.903
**AG**	92	0.57 (0.46-0.86)	0.694	1.41 (1.09-2.22)	0.670	0.09 (0.07-0.12)	
**AA**	34	0.65 (0.48-0.85)		1.55 (1.19-2.43)		0.10 (0.06-0.14)	
**AG/AA**	126	0.60 (0.47-0.86)	0.897	1.47 (1.10-2.23)	0.866	0.09 (0.07-0.12)	0.951
**MAF**	0.42						
**rs8044334**							
**TT**	35	0.66 (0.46-0.89)		1.55 (1.17-2.06)		0.09 (0.06-0.11)	
**GT**	91	0.61 (0.47-0.86)	0.873	1.47 (1.12-2.22)	0.985	0.10 (0.07-0.12)	0.579
**GG**	63	0.59 (0.45-0.91)		1.49 (0.98-2.29)		0.09 (0.07-0.15)	
**GT/GG**	154	0.60 (0.46-0.86)	0.610	1.48 (1.09-2.23)	0.926	0.09 (0.07-0.13)	0.374
**MAF**	0.43						

Continuous variables presented as medians (interquartile ranges)

Table S4. Plasma concentrations of non-provitamin A carotenoids across genotypes of the variants in the BCO1 gene

	n	lutein [μmol/L]	*p*-value	lycopene [μmol/L]	*p*-value	zeaxanthin [μmol/L]	*p*-value
**rs6564851**							
**TT**	89	0.12 (0.09-0.17)		0.37 (0.25-0.47)		0.037 (0.027-0.048)	
**GT**	82	0.13 (0.10-0.17)	0.469	0.39 (0.29-0.49)	0.753	0.034 (0.026-0.047)	0.665
**GG**	18	0.12 (0.08-0.20)		0.39 (0.30-0.50)		0.034 (0.027-0.058)	
**GT/GG**	100	0.13 (0.09-0.17)	0.235	0.39 (0.29-0.49)	0.454	0.034 (0.026-0.049)	0.434
**MAF**	0.31						
**rs7500996**							
**TT**	90	0.13 (0.09-0.17)		0.39 (0.26-0.47)		0.037 (0.027-0.049)	
**CT**	76	0.13 (0.09-0.18)	0.748	0.36 (0.29-0.47)	0.827	0.035 (0.025-0.049)	0.863
**CC**	23	0.11 (0.09-0.16)		0.39 (0.29-0.49)		0.036 (0.025-0.043)	
**CT/CC**	99	0.12 (0.09-0.12)	0.827	0.37 (0.29-0.49)	0.869	0.035 (0.025-0.048)	0.587
**MAF**	0.32						
**rs10048138**							
**GG**	71	0.13 (0.09-0.18)		0.39 (0.30-0.50)		0.036 (0.027-0.052)	
**AG**	92	0.13 (0.09-0.16)	0.789	0.39 (0.27-0.45)	0.499	0.035 (0.025-0.047)	0.938
**AA**	26	0.12 (0.10-0.19)		0.36 (0.24-0.47)		0.036 (0.028-0.050)	
**AG/AA**	118	0.13 (0.09-0.17)	0.619	0.37 (0.25-0.46)	0.299	0.035 (0.026-0.047)	0.878
**MAF**	0.38						

Table S4 (continued). Plasma concentrations of non-provitamin A carotenoids across genotypes of the variants in the BCO1 gene

**rs6420424**							
**GG**	63	0.14 (0.09-0.18)		0.39 (0.26-0.48)		0.037 (0.027-0.055)	
**AG**	92	0.13 (0.09-0.17)	0.803	0.39 (0.29-0.47)	0.875	0.035 (0.025-0.047)	0.648
**AA**	34	0.12 (0.10-0.15)		0.36 (0.24-0.51)		0.036 (0.027-0.053)	
**AG/AA**	126	0.13 (0.09-0.17)	0.517	0.37 (0.28-0.48)	0.895	0.035 (0.027-0.047)	0.380
**MAF**	0.42						
**rs8044334**							
**TT**	35	0.12 (0.09-0.15)		0.36 (0.26-0.45)		0.04 (0.03-0.05)	
**GT**	91	0.13 (0.09-0.18)	0.467	0.36 (0.25-0.47)	0.297	0.04 (0.03-0.05)	0.612
**GG**	63	0.11 (0.08-0.18)		0.40 (0.30-0.50)		0.03 (0.02-0.05)	
**GT/GG**	154	0.13 (0.09-0.18)	0.892	0.39 (0.28-0.49)	0.324	0.04 (0.03-0.05)	0.918
**MAF**	0.43						

Continuous variables are presented as medians (interquartile ranges)
